# Express delivery: dsRNA applications and RNAi nanocarrier systems to combat potato late blight

**DOI:** 10.3389/fpls.2026.1967410

**Published:** 2026-10-19

**Authors:** Ellen Vandenbussche, Kristof De Schutter, Els J. M. Van Damme

**Affiliations:** 1 Laboratory of Biochemistry and Glycobiology, Department Biotechnology, Ghent University, Ghent, Belgium; 2 Molecular Entomology Laboratory, Agrozoology, Department of Plants and Crops, Ghent University, Ghent, Belgium

**Keywords:** nanocarriers, *Phytophthora*, plant-oomycete communication, RNAi, SIGS, silencing, uptake

## Abstract

*Phytophthora* late blight remains one of the most devastating diseases of potatoes, posing persistent challenges to global food security and sustainable agriculture. Although RNA interference (RNAi) has emerged as a powerful and highly specific strategy for pathogen control, its practical application against *P. infestans* has largely remained at the proof-of-concept stage. This limitation does not result from a lack of suitable gene targets, but rather from challenges associated with the delivery, stability, and uptake of RNA molecules under field conditions. In this review, we outline current advances in RNAi-based approaches for the control of *P. infestans*, with a particular focus on spray-induced gene silencing (SIGS) and the potential of nanocarrier-based delivery systems. We summarize proof-of-concept studies targeting essential oomycete genes, discuss the molecular mechanisms underlying double-stranded RNA (dsRNA) uptake in plants and oomycetes, and critically evaluate the strengths and limitations of host-induced gene silencing (HIGS) and SIGS. Unlike recent reviews on nanocarrier-mediated RNA delivery, this manuscript provides a system-specific focus centered on the potato–*P. infestans* pathosystem and the unique biological features of the plant–oomycete interface. Central to this review is the argument that future success of RNAi-based disease management will require systems‑level optimization that integrates target selection, dsRNA design, formulation, delivery, and application timing, while emphasizing that delivery remains a major bottleneck for translation to field applications. We highlight how nanocarriers can enhance dsRNA stability, improve adhesion and cellular uptake, and enable controlled release, thereby transforming SIGS into a robust and environmentally sustainable plant protection strategy. Finally, we position RNAi-based nanobiopesticides within the broader European regulatory and sustainability landscape, emphasizing both regulatory feasibility and the importance of public trust and societal acceptance. Together, these insights position nanocarrier-enabled SIGS as a promising cornerstone of next-generation, sustainable late blight management.

## 1 Introduction

*Phytophthora infestans*, the oomycete causing late blight, remains one of the most destructive crop pathogens worldwide, particularly under cool and humid conditions (Wang et al., 2025b). Late blight is associated with substantial yield losses and annual economic damage exceeding 6 billion USD globally (González-Jiménez et al., 2023; Wang et al., 2025b). In Europe, field studies estimate that *P. infestans* is responsible for 8% of the annual potato yield loss in Ireland (Cucak et al., 2021). It also causes severe yield reductions during epidemic years in southern Sweden (González-Jiménez et al., 2023). Although global loss estimates vary among regions and growing seasons, late blight is consistently ranked among the most economically damaging diseases of solanaceous crops, especially in production systems where weather favors repeated infection cycles (Ferdus et al., 2025).

For decades, late blight management has relied primarily on chemical fungicides, including phenylamides, organochlorines, quinone‑outside inhibitors (QoIs), and carboxylic acid amides, which are often applied in intensive preventive and curative spray programs (Ivanov et al., 2021). While these compounds remain indispensable tools, their extensive use of has raised concerns regarding environmental contamination and residue accumulation, off-target impacts, and declining efficacy due to resistance selection (Ivanov et al., 2021; Dong and Zhou, 2022). These concerns are particularly problematic for *P. infestans*, as late blight epidemics can progress rapidly and often necessitate frequent fungicide applications, thereby increasing both production costs and selection pressure. Additionally, late blight management is complicated by the pathogen’s clonal population structure, strong adaptive potential, and the recurrent emergence of fungicide-insensitive or resistant lineages (Kvick Nastaj, 2023; Coomber et al., 2024; Wang et al., 2025b).

Because of these challenges, modern plant protection research has increasingly shifted toward biologically based and molecularly precise alternatives. Among these, RNA interference (RNAi)-based approaches have emerged as a promising strategy due to their high specificity, environmental compatibility, and flexibility in target selection. RNAi is a conserved gene-silencing mechanism in which double-stranded RNA (dsRNA) molecules trigger sequence-specific degradation of messenger RNA (mRNA), thereby suppressing gene expression at the post-transcriptional level (Yang et al., 2000; Agrawal et al., 2003). In the concept of crop protection, RNAi enables selective targeting of genes that are essential for survival, metabolism, or virulence of the pathogen, while minimizing off-target effects on plants, beneficial organisms, and humans (Zhang et al., 2017).

Two main deployment strategies have been explored for the control of *P. infestans*: host-induced gene silencing (HIGS) and spray-induced gene silencing (SIGS) (Ivanov et al., 2021; Padilla-Roji et al., 2023). HIGS relies on transgenic plants engineered to produce pathogen-targeting RNA molecules, providing sustained RNA expression but requiring stable genetic transformation and facing considerable regulatory and breeding challenges (Schlemmer, 2022). In contrast, SIGS involves the exogenous application of dsRNA to the plant surfaces, offering greater flexibility and a potentially more acceptable route for near-term field use. Because an RNAi strategy can be adapted quickly, SIGS may enable faster responses to emerging pathogen variants without the need to develop new transgenic cultivars. However, the efficacy of SIGS under field conditions is challenged by the limited environmental stability of naked dsRNA and its variable uptake by plants and *P. infestans* cells (Mclaughlin et al., 2023; Xi et al., 2025).

To address these limitations, nanocarrier-based delivery systems have emerged as a particularly powerful tool for enhancing RNA-based plant protection. While several recent studies have outlined general frameworks for rational dsRNA design, scalable production, and nanodelivery, these reviews did not examine how these concepts intersect with the specific biological constraints of the potato–*P. infestans* interaction. In contrast, the present review integrates nanodelivery principles with the mechanistic realities of dsRNA transport, uptake, and stability at the plant–oomycete interface. Recent developments in plant nanobiotechnology show that engineered nanomaterials can act as highly effective dsRNA delivery platforms owing to their tunable physical and chemical properties, and their ability to interact intimately with biological surfaces (Xi et al., 2025). Through electrostatic interactions, hydrogen bonding, or hydrophobic forces, nanocarriers can form stable complexes with dsRNA, shielding it from rapid enzymatic degradation and environmental breakdown (Christiaens et al., 2020a; Mat Jalaluddin et al., 2023). In addition to enhancing stability, many nanocarriers improve foliar adhesion, facilitate penetration through the plant cuticle, and promote cellular uptake in both plant and pathogen tissues—often through endocytic pathways and improved early endosomal escape (Kandhol et al., 2023; Mamun et al., 2025). These combined effects substantially increase the persistence and bioavailability of dsRNA after spraying. As a result, nano-enabled SIGS is considered as a viable route toward more durable, targeted, and field-deployable late blight management, especially under conditions where naked dsRNA would otherwise exhibit poor stability or limited uptake.

## 2 dsRNA application against *Phytophthora*

### 2.1 RNAi machinery

RNAi is an evolutionarily conserved gene-silencing mechanism that protects eukaryotic genomes from viruses, transposable elements, and other invasive nucleic acids (Obbard et al., 2009). The phenomenon was first described in *Caenorhabditis elegans*, where dsRNA was shown to trigger potent, sequence-specific gene silencing far more efficiently than either sense or antisense RNA alone (Fire et al., 1998). This discovery was built on earlier observations of homology-dependent gene silencing in plants and fungi, including co-suppression in petunia and antiviral silencing in plant–virus interactions, which had already revealed the systemic and sequence-specific nature of RNA-based defense mechanisms (Napoli et al., 1990; Lindbo et al., 1993). Together, these studies established RNAi as a highly conserved eukaryotic defense strategy.

Across eukaryotes, RNAi is initiated when endogenous or exogenous dsRNA precursors—derived from viral replication intermediates, transposons, hairpin RNAs, or bidirectionally transcribed loci—are detected in the cytoplasm (Geley and Muller, 2004). These dsRNA molecules are processed by Dicer, an RNase III enzyme that cleaves dsRNA into 21–26 nt RNAs, including small interfering RNAs (siRNAs) and micro RNAs (miRNAs) (Macrae et al., 2006). Following cleavage, the siRNA duplex is loaded onto an Argonaute (AGO) protein within the RNA-induced silencing complex (RISC), where the passenger strand is removed, leaving a single-stranded guide RNA that directs the RISC to complementary mRNA targets for cleavage or translational repression (Nakanishi, 2016). In many organisms, RNA-dependent RNA polymerases (RdRPs) further amplify the silencing signal by converting target RNAs into secondary dsRNA, thereby generating additional siRNAs and reinforcing systemic or transgenerational silencing responses (Dalmay et al., 2000; Sijen et al., 2001). Beyond post-transcriptional silencing, RNAi pathways can also induce transcriptional gene silencing (TGS) through chromatin modification, including deposition of repressive histone marks and heterochromatin formation, enabling long-term or heritable epigenetic regulation (Braun, 2024).

Although oomycetes are phylogenetically distinct from fungi, *Phytophthora* species possess a complete and functional RNAi machinery that is highly conserved with those of plants and fungi. Early work identified genes encoding Dicer-like (DCL), PiAGO, PiRdRP, and chromatin-associated silencing proteins in *P. infestans*, and provided functional evidence that these components participate in gene silencing (Vetukuri et al., 2011a). Subsequent studies showed that *P. infestans* upregulates *PiDcl2* and *PiRdr1* in response to viral infection, supporting the existence of an antiviral RNAi response analogous to those described in plants and fungi (Mascia et al., 2019). Comparative analyses across *Phytophthora* species further highlight both the conservation and functional diversification of RNAi components. In *P. capsici*, DCL1, DCL2, Exportin-5, RdR, and six AGO proteins have been identified, with expression patterns varying depending on host context, suggesting dynamic regulation of RNAi pathways during infection (Sevillano-Serrano et al., 2026). Similarly, *P. sojae* encodes conserved AGO, Dicer-like, and RdRP proteins and produces abundant 24–26 nt small RNAs associated with genome-defense functions, such as transposon silencing and suppression of repetitive DNA activity, suggesting their role in maintaining genome stability (Wang et al., 2020, Wang et al., 2026a). Across oomycetes, RNA silencing is known to restrict transposon expansion, and several biocontrol species, such as *Pythium oligandrum*, *Pythium periplocum*, and *Lagenidium giganteum*, produce mobile miRNA-like RNAs (milRNAs) capable of targeting genes in prey organisms, implying that cross-kingdom RNA trafficking contributes to ecological interactions and pathogenicity. Consistent with this hypothesis, milRNAs from these species were also predicted to target transcripts in *P. infestans*, suggesting a potential trans-boundary regulatory role (Piombo et al., 2023). Despite these advances in our understanding of oomycete RNAi pathways, transcriptional gene silencing remains poorly characterized in *P. infestans*. Understanding how RNA-based silencing regulates genome stability, effector gene expression, and host adaptation represents a major frontier in oomycete biology.

SIGS exploits the endogenous RNAi machinery of both plants and pathogens, offering a transgene-free approach to disease control. Following application to plant surfaces, exogeneous dsRNA can enter plant tissues, although the cellular and extracellular routes responsible for its uptake remain incompletely understood. Once internalized, dsRNA may be processed by plant Dicer-like proteins into primary siRNAs, which are subsequently loaded into AGO-containing RISCs and guide the sequence-specific cleavage or translational repression of complementary transcripts, including pathogen-derived mRNAs. This canonical pathway is well established in plant antiviral defense and developmental regulation. Plant RdRP-mediated amplification generally requires a complementary single-stranded RNA template. Consequently, pathogen-targeting dsRNA does not necessarily provide a suitable substrate for transitive amplification in the host plant. Although a *Fusarium*–wheat SIGS study suggested that pathogen- derived siRNAs may undergo amplification within plant tissues, canonical RdRP activity has not been demonstrated in potato SIGS against *P. infestans*. Therefore, while the involvement of non-canonical or template-independent RdRP amplification pathways cannot be excluded, it remains speculative at present (Song et al., 2018).

Recent mechanistic studies further indicate that foliar-applied dsRNA can remain largely unprocessed and move systemically through the plant apoplast, reaching vegetative, reproductive, and below-ground tissues (Brosnan et al., 2026). These findings suggest that long-distance RNA transport is not limited to Dicer-derived siRNAs and may involve multiple parallel pathways, including the movement of intact dsRNA through the apoplast. While systemic transport of endogenous plant small RNAs through the phloem has been described extensively (Vance and Vaucheret, 2001; Biedenkopf et al., 2020), the extent, stability, and molecular determinants of intact dsRNA movement remain poorly understood.

When *P. infestans* colonizes treated tissues, RNA transfer from the host plant to the pathogen may occur through several potential routes. While extracellular vesicle (EV)-mediated delivery has been demonstrated in plant–fungus interactions, its contribution in plant–oomycete systems remains unestablished. In addition, *P. infestans* can take up externally supplied dsRNA, although uptake efficiency is limited and varies between cell types and developmental stages (Qiao et al., 2021). Fluorescence-based studies have detected dsRNA within *P. infestans* mycelia and sporangia, supporting pathogen-side internalization and suggesting that at least part of the silencing process occurs within the pathogen. However, the relative contribution of direct uptake from the extracellular environment, transfer through plant cells or apoplastic fluids, and vesicle-associated delivery remain unresolved. Therefore, observations from fungal systems, including EV-mediated transfer of plant small RNAs, should not be extrapolated directly to oomycetes without experimental validation (Cai et al., 2018).

Once inside *P. infestans*, dsRNA or derived siRNAs may be processed by the pathogen’s endogenous RNAi machinery and incorporated into pathogen RISCs, ultimately reducing the abundance of complementary target transcripts. Nevertheless, the precise sequence of events underlying SIGS should be interpreted with caution, as evidence supporting individual steps, including uptake, intracellular processing, inter-kingdom transfer, and target-transcript degradation varies considerably among pathosystems and experimental settings. Thus, SIGS is best viewed as a multistep process that may involve multiple parallel uptake and transport routes, in which RNA stability, plant uptake and systemic movement, pathogen internalization, intracellular processing, and gene silencing efficacy are influenced by factors such as plant tissue type, pathogen developmental stage, RNA format, and environmental conditions. The currently proposed routes of SIGS-mediated defense against oomycetes are summarized in **Figure 1**.

**Figure 1:**
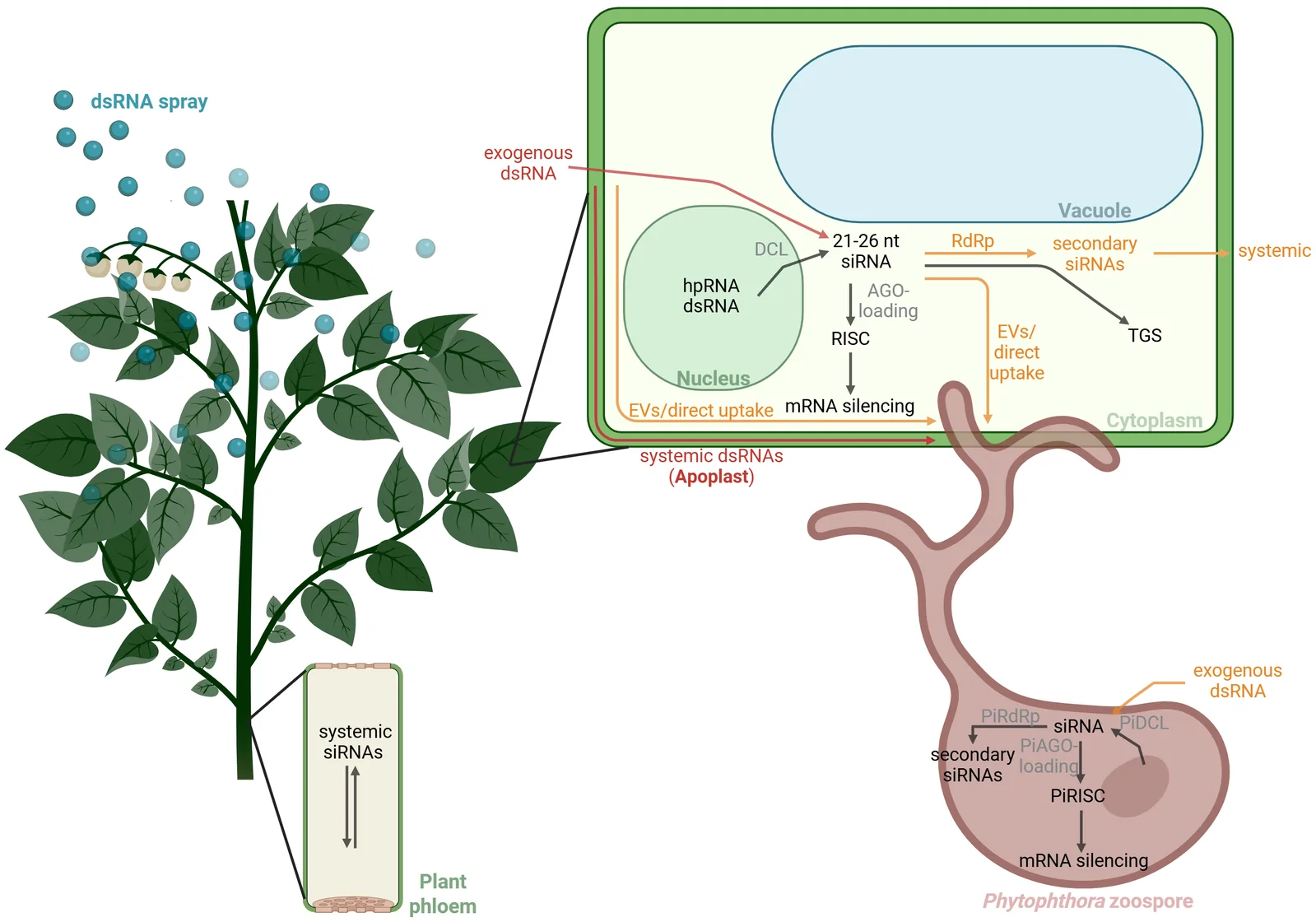
Schematic overview of the RNAi pathway activated by exogenous dsRNA in potato during SIGS against *Phytophthora*. The figure illustrates both the plant and oomycete intrinsic RNAi cascade, as well as the cross-kingdom RNA transfer that underpins SIGS-mediated control of late blight. Transport steps are differentiated according to their level of experimental support: black arrows indicate experimentally demonstrated routes, red arrows indicate supported but indirect evidence, and orange arrows represent hypothesized mechanisms such as extracellular vesicle (EV)-mediated RNA transfer to *P. infestans*. Created in https://BioRender.com.

### 2.2 Evidence for dsRNA susceptibility: HIGS vs. SIGS

Multiple proof-of-concept studies have clearly demonstrated that *P. infestans* is highly susceptible to dsRNA-mediated gene silencing, and that both HIGS and SIGS can efficiently down-regulate effector, metabolic and developmental genes. HIGS relies on the stable integration of hairpin or dsRNA constructs into the plant genome, enabling the host to constitutively produce dsRNA targeting essential pathogen genes throughout its life cycle (Zand Karimi and Innes, 2022). These dsRNAs are processed into siRNAs by plant Dicer-like enzymes and can subsequently be transported to the infection sites, where they are transferred into invading *P. infestans* cells and engage the pathogen’s endogenous RNAi machinery to induce sequence-specific silencing. Several transgenic systems have demonstrated that plant-derived dsRNA can effectively reduce *P. infestans* gene expression and disease severity under laboratory and greenhouse conditions (**Table 1**).

**Table 1:** Summary of published studies investigating host-induced gene silencing (HIGS) and spray-induced gene silencing (SIGS) for the control of *Phytophthora* species.

Organism	Application method	Experimental system	Target gene(s)	dsRNA fragment length	Main effect	mRNA transcript level quantified	Reference
*P. infestans*	*in vitro* delivery	protoplasts, mycelium	*Gfp* *Inf1* *Cdc14*	199 bp 260 bp 167 bp	reducing transcript levels and altering growth/development	yes	(Whisson et al., 2005)
*P. infestans*	pathogen-internal transgene-mediated RNAi	spores	*Inf1*	376 bp	hairpin transgenes efficiently triggered siRNA production and robust silencing	no	(Ah-Fong et al., 2008)
*P. infestans*	pathogen-internal transgene-mediated RNAi	potato detached leaf assay	*PiAvr3a*	/	fusion to the short, interspersed element resulted in strong, stable silencing, abolishing effector expression and modifying virulence	no	(Vetukuri et al., 2011b)
*P. parasitica*	pathogen-internal transgene-mediated RNAi, HIGS	Arabidopsis detached leaf and whole plant assay	*Gfp* *PnPma1*	612 bp 262 bp	Neither pathogen-internal dsRNA expression nor HIGS triggered gene silencing	yes	(Zhang et al., 2011)
*P. infestans*	HIGS	potato whole plant assay	*Avr3a*	444 bp	reduced effector transcript levels during infection and partial resistance, with delayed symptom development and lower disease severity	yes	(Sanju et al., 2015)
*P. infestans*	HIGS	potato whole plant assay	*Gfp* *PiGpb1* *PiCesa2* *PiPec* *PiGadph*	441 bp 491 bp 451 bp 479 bp 155 bp	constructs with *PiGpb1* showed significantly reduced pathogen growth and symptom severity	yes	(Jahan et al., 2015)
*P. infestans*	*in vitro* delivery, SIGS	spores, mycelium, potato detached leaf assay	*Gfp* *PiGpb1* *PiOsbp* *PiHmp1* *PiCut3* *PiEndo3*	436 bp 332 bp / / / /	reduced lesion size and sporulation, with efficacy strongly depending on the target gene and dsRNA length	yes	(Kalyandurg et al., 2021)
Several pathogens including *P. infestans*	*in vitro* delivery, SIGS	spores, mycelium, potato detached leaf assay	*Yfp* *PiDcl1* *PiHmp1 PiGpb1* *PiDctn1+Sac1*	720 bp 238 bp 229 bp 146 bp /	poor uptake and silencing effects of naked dsRNA	yes	(Qiao et al., 2021)
*P. infestans*	*in vitro* delivery, SIGS	mycelium, potato detached leaf and whole plant assay	*PiSdh* *PiHsp90* *PiEf1-α* *PiPld-3* *PiGpi-Ham34*	1030 bp 425 bp 402 bp 563 bp 423 bp	multigene dsRNA sprays significantly reduced symptom severity compared with single target dsRNA treatment	yes	(Sundaresha et al., 2022)
*P. infestans*	SIGS	potato whole plant assay	*Inf1* *Inf4*	496 bp 435 bp	suppressed elicitin expression and significantly reduced pathogenicity, with smaller lesions and decreased sporulation	no	(Ivanov and Golubeva, 2023)
*P. capsici*	leaf infiltration	tobacco whole plant assay	*PcNlp2* *PcNlp6*	400 bp 540 bp	reduced infection and necrosis	yes	(Park et al., 2023)
*P. cinnamomi*	pathogen-internal transgene-mediated RNAi	*Castanea sativa* root inoculation	*Npp1*	519 bp	reduced protein accumulation and attenuated necrosis on host plants	no	(Pascoal-Ferreira et al., 2023)
*P. infestans*	*in vitro* delivery, SIGS	spores, mycelium, potato detached leaf assay	*Gfp* *PiGpb1* *PiOsbp* *PiHmp1* *PiCut3 PiEndo3*	Short: 200–400 bp Long: 700 bp	Inhibition mycelial growth and significantly reduced disease progression, no conclusion on dsRNA fragment size	yes	(Sundararajan, 2023)
*P. infestans*	HIGS	potato detached leaf and whole plant assay, potato plants in the field	*Als*	300 bp	strongly enhanced resistance, with greatly reduced lesion development and sporulation	yes	(Temme et al., 2025)
*P. infestans*	*in vitro* delivery	spores, mycelium	*Gfp*	546 bp	low dsRNA uptake by spores and mycelium	no	(Kostov and Smagghe, 2026)
*P. infestans*	SIGS	potato whole plant assay	*PiGpb1* *PiCut3*	664 bp/	SIGS little effect on the relative abundance of dominant native potato phyllosphere bacteria and fungi	no	(Ghosh et al., 2026)

The table compiles target organism and target genes, delivery strategies, dsRNA fragment length, and reported levels of disease suppression, highlighting the development of RNAi-based technologies for oomycete management and the emerging evidence for cross-kingdom RNA transfer.

Several studies demonstrated that *Phytophthora* spp. can process dsRNA internally. Ah-Fong et al. (2008) were the first to show that pathogen-internal hairpin constructs expressed in *P. infestans* spores efficiently produced siRNAs and robustly silenced the elicitin *Inf1*. Vetukuri et al. (2011b) expanded this concept by generating *P. infestans* strains transformed with *P. infestans avirulence protein 3a* (*PiAvr3a*), an Arg-X-Leu-Arg (RXLR) effector that suppresses host immunity, thereby enabling pathogen-internal production of dsRNA. The concept pathogen-internal RNAi has also been explored in other *Phytophthora* species. In *P. parasitica*, pathogen-internal dsRNA expression did not induce silencing (Zhang et al., 2011), highlighting species-specific differences in RNAi responsiveness. In *P. cinnamomi*, transgene-mediated silencing of *necrosis inducing protein 1* (*Npp1*) reduced protein accumulation and attenuated necrosis on *Castanea sativa* roots (Pascoal-Ferreira et al., 2023), demonstrating that pathogen-internal RNAi can modulate virulence traits beyond *P. infestans*. Together, these studies show that *Phytophthora* spp. possess a functional RNAi machinery capable of processing dsRNA internally.

Building on this mechanistic foundation, subsequent studies applied HIGS to target RXLR effectors from the plant side. Transgenic potato lines expressing a 444-bp hairpin dsRNA construct targeting *PiAvr3a*, showed reduced effector transcript levels during infection and partial resistance, including delayed symptom development and lower disease severity (Sanju et al., 2015). HIGS was subsequently extended to additional *P. infestans* genes involved in growth and pathogenicity. Jahan et al. (2015) generated hairpin constructs targeting *green fluorescent protein* (*Gfp*) and *P. infestans G-protein beta subunit 1* (*PiGpb1*), *cellulose synthase A2* (*PiCesa2*), *pectinesterase* (*PiPec*), and *glyceraldehyde-3-phosphate dehydrogenase* (*PiGadph*) in potato plants. Among these, silencing *PiGpb1* in particular caused impaired hyphal growth, altered morphology, reduced invasive capacity, and diminished lesion expansion. These results demonstrate that targeting housekeeping and pathogenicity-related genes through HIGS can substantially compromise pathogen development and infection success. Notably, HIGS has also been applied to target core metabolic genes. For example, field experiments using transgenic potato lines expressing hairpins targeting the *P. infestans* acetolactate synthase (*Als*) gene—a key enzyme in branched-chain amino acid biosynthesis—demonstrated substantially enhanced resistance to late blight under natural infection pressure. These plants exhibited markedly reduced lesion development and sporulation, indicating that silencing of a central metabolic enzyme can compromise pathogen fitness and epidemic potential (Temme et al., 2025). Collectively, these studies illustrate that RNAi-mediated knockdown of effectors, signaling components and metabolic enzymes attenuates virulence, restricts growth and reduces sporangial production. This provides strong functional validation for the effectiveness of RNAi-based gene-silencing in *P. infestans* and highlights its value as a powerful tool for functional genomics.

Despite these promising results, several key limitations restrict the broad agricultural deployment of HIGS. Transformation of potato and other major crops remains labor-intensive, highly genotype-dependent, and technically challenging, restricting the use of HIGS to a relatively limited number of cultivars and hindering iterative target validation (Nagle et al., 2018). Furthermore, although the generation of multiple siRNAs from a single dsRNA molecule makes resistance through mutations in the targeted *P. infestans* transcript less likely, prolonged and uniform selection pressure may still favor the emergence of RNAi insensitivity. Such resistance could potentially involve changes in *P. infestans* dsRNA uptake mechanisms or other components of the RNA silencing pathway, as demonstrated in the Colorado potato beetle where impaired dsRNA uptake resulted in broad insensitivity to RNAi triggers, rather than target-specific resistance (Yin and Hulbert, 2015; Mishra et al., 2021). These scientific and logistical challenges are compounded by stringent regulatory frameworks governing the application of genetically modified crops and by limited public acceptance of GMOs, particularly in Europe, creating substantial legal, economic and societal barriers to large-scale deployment of HIGS-based potato varieties (Bawa and Anilakumar, 2013; Eriksson et al., 2018; European Parliamentary Research Service (Eprs), 2022).

SIGS offers a complementary, non-transgenic avenue for exploiting RNAi against *Phytophthora* through the exogenous application of dsRNA, most commonly via foliar sprays. In this approach, dsRNA or siRNA molecules are applied directly to plant surfaces, where they can be taken up by plant tissues and/or the pathogen itself before being processed by the respective RNAi machinery to induce sequence-specific gene silencing. Because SIGS does not require genetic modification of crops, it circumvents many of the technical, regulatory and societal barriers associated with HIGS and fits more readily within conventional crop protection programs (Hoang et al., 2022; Mannaa et al., 2026).

Mechanistic studies with *Phytophthora* species provided evidence that exogenously applied dsRNA can be taken up by the pathogen and processed into siRNAs. Early *in vitro* experiments demonstrated that naked dsRNA fragments of several hundred base pairs targeting *green fluorescent protein* (*Gfp)*, elicitor *Inf1*, and *cell division cycle 14* (*Cdc14)*, introduced into *P. infestans* protoplasts and mycelia, induced transient and sequence-specific knockdown of endogenous genes, resulting in measurable effects on growth and development of the pathogen (Whisson et al., 2005). These findings provided key proof that the pathogen’s RNAi machinery can be engaged by external dsRNA and that the resulting phenotypic effects, including impaired hyphal growth and altered morphology are consistent with those observed in HIGS systems. Subsequent studies applied this concept to foliar treatments on potato plants. Preventive spraying of dsRNAs targeting genes such as *P. infestans G-protein beta subunit* (*PiGpb1*), *oxysterol binding protein* (*PiOsbp*), *cutinase* (*PiCut3*), *haustorial membrane protein* (*PiHmp1*) and *endo-1, 3(4)-b-glucanase* (*PiEndo3*) reduced sporulation *in vitro* and lesion size in potato leaves, demonstrating that exogenous dsRNA can suppress late blight symptoms without the need for plant transformation (Kalyandurg et al., 2021). More recent studies have refined the SIGS approach by highlighting the critical roles of pathogen dsRNA uptake and target gene selection in determining silencing efficiency. For example, dsRNA sprays directed against the elicitin genes *inf1* and *inf4* reduced transcript abundance and significantly suppressed pathogenicity on potato, resulting in smaller lesions and lower sporulation rates (Ivanov and Golubeva, 2023). Together with studies targeting RXLR effectors, these findings indicate that exogenous silencing of endogenous genes can effectively slow down disease progression.

The potential of SIGS is further supported by studies showing that efficacy depends on both dsRNA design and pathogen target selection. In *P. infestans*, foliar application of gene-specific dsRNAs enabled pathogen uptake and reduced expression of selected target genes and disease progression, although the magnitude of the effect varied among targets (Kalyandurg et al., 2021; Qiao et al., 2021). Sundararajan (2023) investigated dsRNA constructs of approximately 200–400 bp and 700 bp, but their results did not establish a universal optimal dsRNA length or demonstrate that shorter fragments consistently provide stronger uptake or silencing. In addition, multigene dsRNA spray formulations have substantially suppressed mycelial growth and disease development in controlled environments while enabling the simultaneous silencing of multiple pathogen genes, thereby reducing the likelihood of resistance development (Sundaresha et al., 2022).

From a practical perspective, SIGS offers several advantages over HIGS. Target sequences can be redesigned rapidly as new pathogen strains or resistance mechanisms emerge, eliminating the need to generate new transgenic plants and enabling more agile responses to changing pathogen populations (Hoang et al., 2022; Mannaa et al., 2026). Furthermore, dsRNA formulations can be applied using existing agricultural infrastructure and readily incorporated into existing spray programs. Because dsRNA molecules are naturally occurring and inherently biodegradable, they are degraded by environmental RNases into harmless nucleotides, typically within days, providing a favorable environmental profile compared with many conventional chemical fungicides (Bachman et al., 2020; Rodrigues et al., 2021).

Nevertheless, significant challenges limit the effectiveness of SIGS under field conditions (**Figure 2**). Naked dsRNA is highly susceptible to degradation by ultraviolet (UV) radiation and nucleases, and its intrinsic instability in the phyllosphere causes the dsRNA to become undetectable within hours after application (Bachman et al., 2020; Christiaens et al., 2020b; Zarrabian and Sherif, 2025). Exposure to UV radiation causes photochemical cleavage of RNA strands, while environmental nucleases originating from microbial communities can rapidly degrade dsRNA upon deposition (Peng et al., 2018; Parker et al., 2019). In addition, rainfall and irrigation can wash off the dsRNA before it is absorbed by plant tissues or the pathogen, and temperature fluctuations can further accelerate hydrolysis (Bachman et al., 2020; Romeis and Widmer, 2020). Moreover, dsRNA uptake by plants and *P. infestans* is often inefficient, meaning that only a fraction of the applied dsRNA reaches the pathogen’s RNAi machinery in sufficient quality and quantity to induce robust silencing (Bennett et al., 2020; Hoang et al., 2022). Although *P. infestans* possesses a fully functional intracellular RNAi machinery, the uptake of externally applied dsRNA remains limited. Once internalized, dsRNA is efficiently processed and can trigger strong, sequence-specific gene silencing, demonstrating that the pathogen is intrinsically RNAi-competent. Therefore, RNAi susceptibility should be distinguished from SIGS efficacy, which is primarily constrained by poor dsRNA delivery. Consistent with this, Qiao et al. (2021) reported that uptake of naked dsRNA is limited and varies considerably among cell types and developmental stages, indicating that delivery rather than RNAi competence represents the limiting step for effective SIGS in *P. infestans*. As a result, some naked dsRNA treatments produce only modest silencing despite the presence of intact RNAi machinery in both the plant and the pathogen. These limitations help to explain why the strongest SIGS effects have generally been observed under controlled conditions rather than in the field. Consequently, successful field-scale translation will require improved formulation, such as carriers or protective matrices, or enhanced delivery strategies. Addressing these challenges through improved dsRNA stability, optimized fragment design and target selection, and more efficient delivery to both plant and pathogen will be essential to move SIGS from a promising experimental strategy to a reliable, field-ready technology for late blight management.

**Figure 2:**
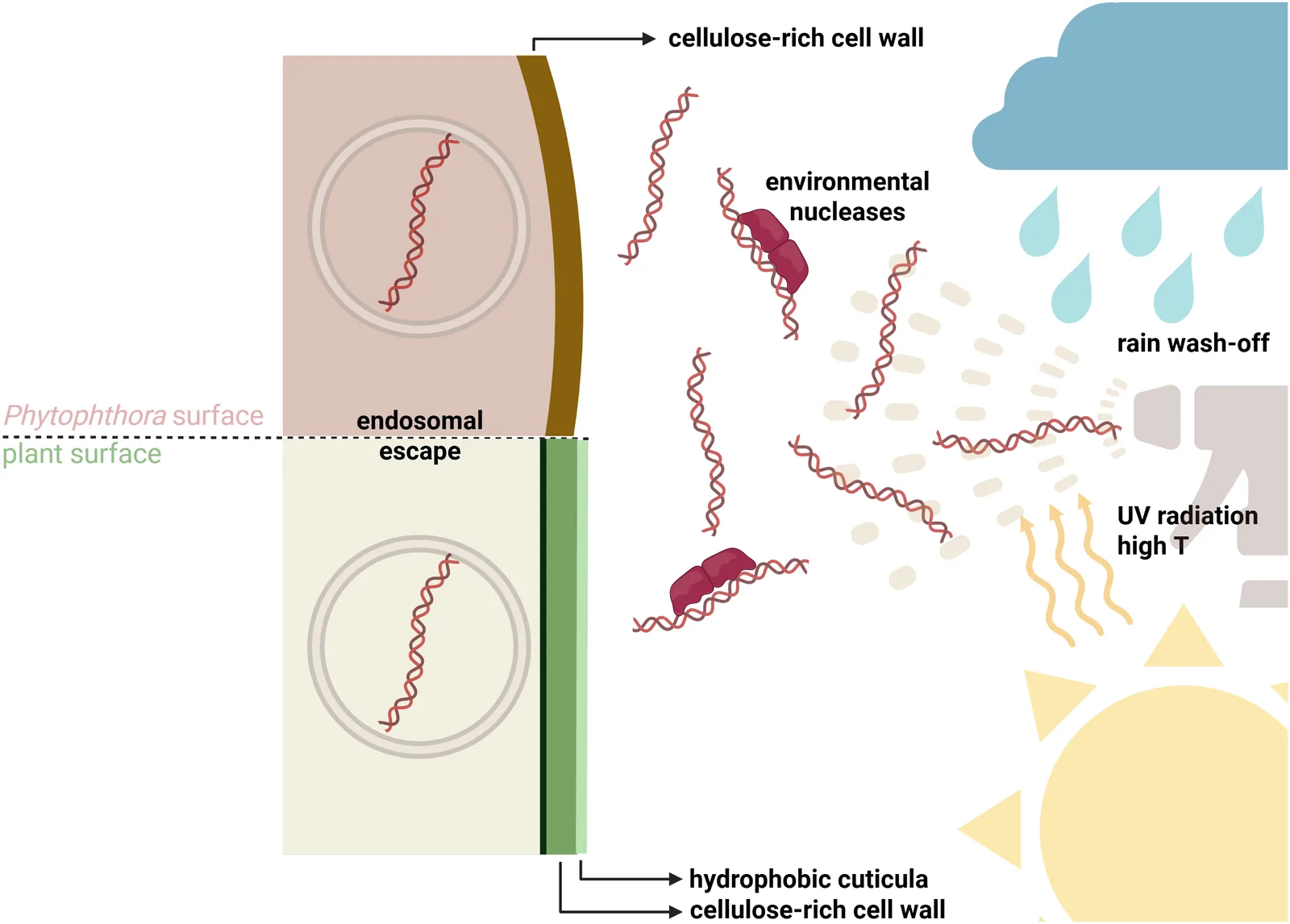
Major barriers limiting the efficacy of naked dsRNA sprays against *Phytophthora*. The figure summarizes the environmental, physical, and cellular obstacles that prevent exogenously applied dsRNA from reaching its RNAi targets. Naked dsRNA is rapidly degraded by UV radiation, temperature fluctuations, and environmental nucleases, and is easily washed off the hydrophobic leaf surface. Surviving molecules face additional barriers, including the plant cuticle, the cellulose -rich cell walls of both plant tissues and *Phytophthora*, and inefficient cellular uptake. Together, these multilayered constraints explain the short persistence and limited silencing efficiency of naked dsRNA in SIGS applications. Created in https://BioRender.com.

## 3 Nanocarrier-based solutions for RNAi delivery in *P. infestans*

### 3.1 Rationale for nanocarrier use

SIGS has gained considerable attention as a non-transgenic RNA-based plant protection strategy, yet the practical application of naked dsRNA remains severely limited by its poor stability, inefficient uptake, and short-lived biological activity (Romeis and Widmer, 2020; Mendoza-Alatorre et al., 2025). Although naked dsRNA can suppress pathogens such as *P. infestans* under controlled conditions, its protective effect typically persists for only a few days, largely because most dsRNA is rapidly lost through UV-induced cleavage, nuclease degradation, wash-off, and temperature-driven hydrolysis. As a result, much of the applied dsRNA is lost before it can interact with plant or pathogen cells, resulting in a limited and inconsistent protective window.

Even when dsRNA avoids immediate degradation and remains on the leaf surface long enough to interact with the plant or pathogen cells, its uptake into plant tissues or *P. infestans* structures remains inefficient. The plant cuticle forms a hydrophobic barrier that restricts retention and penetration of aqueous dsRNA solutions (Bennett et al., 2020; Hoang et al., 2022). Although surfactants can improve leaf coverage, they may simultaneously destabilize RNA molecules or interfere with uptake mechanisms (Bennett et al., 2020). Beneath the cuticle, the cellulose-rich cell walls of both plant epidermal cells and oomycete hyphae impose an additional physical barrier that restricts the passage of large, negatively charged dsRNA molecules (Bennett et al., 2020; Yilmaz et al., 2020; Naka et al., 2023). Furthermore, unlike some animals, plants and oomycetes lack efficient dsRNA transport mechanisms, meaning that internalization largely relies on slow and low-efficiency endocytosis processes. Consequently, only a small proportion of the applied dsRNA reaches the cytoplasm, where RNAi is initiated. Even when cellular uptake occurs, the resulting RNAi response is transient because the limited amount of internalized dsRNA is rapidly processed into siRNAs and subsequently diluted through cell division, tissue expansion, or pathogen growth (Bartlett and Davis, 2006). Together, these limitations explain why naked dsRNA has yet to progress beyond experimental trials, despite the strong mechanistic evidence supporting RNAi-based pathogen control.

To address these limitations, a wide range of nanocarrier-based delivery systems have emerged as a promising and versatile solution for enhancing SIGS efficacy. By encapsulating or electrostatically complexing dsRNA, nanocarriers protect RNA molecules from UV radiation, nuclease activity, and temperature-induced instability, thereby extending their persistence on leaf surfaces and within plant tissues (Yang et al., 2022; Kandhol et al., 2023). Improved adhesion further reduces wash-off during rainfall or irrigation, ensuring prolonged dsRNA availability at the application site. Importantly, nanocarriers can facilitate cellular uptake by promoting endocytosis, enabling cuticle penetration, and overcoming cell wall size-exclusion limits (Ma et al., 2022; Wang et al., 2026b). These properties substantially increase the amount of dsRNA reaching the cytoplasm of plant or pathogen cells, enabling efficient gene silencing at lower application doses.

In addition to improving stability, nanocarriers can be engineered to provide controlled or sustained release of dsRNA, ensuring a continuous supply of silencing triggers and prolonging the duration of protection well beyond the capabilities of naked dsRNA (Moorlach et al., 2026). Surface functionalization of the carrier offers opportunities to optimize delivery performance. For example, cationic groups, such as primary amines present on chitosan, polymers, or dendrimers, strengthen electrostatic interactions with the dsRNA, protect it from nucleases, and improve foliar adhesion (Mitter et al., 2017; Wang et al., 2023d; Vereecken et al., 2026). Protonatable amines can also promote endosomal escape via the proton-sponge effect, thereby increasing cytosolic release following endocytosis (Benjaminsen et al., 2013). These tunable functional groups provide a direct means to optimize dsRNA delivery efficiency for SIGS applications. Furthermore, their modular design enables the co-delivery of multiple dsRNAs targeting different pathogen genes, allowing synergistic silencing and reducing the likelihood of resistance development (Yong et al., 2021). Through these combined functionalities, nanocarrier-based delivery systems have the potential to transform SIGS from a transient, environmentally constrained approach into a robust, durable, and field-compatible plant protection strategy. As such, nanotechnology is increasingly regarded as a key enabling component for translating RNAi-based crop protection from laboratory proof-of-concept to practical agricultural application.

### 3.2 Overview of nanocarrier platforms applied to SIGS against *P. infestans*

A growing body of research demonstrates that nanocarriers can substantially enhance the stability, uptake, and biological performance of dsRNA for SIGS-based control of *P. infestans*. Both synthetic and biodegradable delivery platforms have been explored, each offering distinct advantages in terms of dsRNA and nucleic acid protection, delivery efficiency, and environmental compatibility (**Table 2**).

**Table 2:** Summary of published studies investigating nanocarrier- and delivery vehicle–mediated application nucleic acid delivery for the control of *Phytophthora* species.

Carrier type	Synthetic/bio-degradable	Target organism	Experimental system	Target gene(s)	Dose	Complex information	Main effect	Transcript quantification	Reference
Star polymer (SPc)	synthetic	*P. infestans*	potato plants in greenhouse	chitosan (CSC) or eugenol (ESC) (no dsRNA)	0.5 g/L CSC, 0.15% ESC	volume ratio 1:1 (CSC or ESC), 17.40 nm, cationic	amplified plant defense after 12 h and improved late-blight suppression at 6 dpi by 30-40% compared to chitosan or eugenol alone	defense-related transcripts e.g. PR1, WRKY34): 4.7-fold change	(Wang et al., 2021)
			potato plants in the field		1250 g/ha CSC, 3.75 L/ha ESC		48.9% protection for CSC at 21 dpi and 69.3% for ESC at 26 dpi	no	
Nanoclay	inorganic, naturally occurring, non-biodegradable	*P. infestans*	*in vitro*	*PiSdh* *PiHsp90* *PiEf1-α* *PiPld-3* *PiGpi-Ham34*	naked dsRNA: 100–500 ng/µL	/	multi-gene targeting showed 70-90% mycelial growth reduction compared to naked dsRNA at 10 dpi	no	(Sundaresha et al., 2022)
			potato detached leaf assay		200 ng/µL dsRNA, 2–50 ppm nanoclay	/	Reduced lesion development, symptom severity and sporulation relative to naked dsRNA using 10 ppm nanoclay	no	
			potato whole plant assay		250 ng/µL dsRNA, 2–50 ppm nanoclay	/	Disease severity 4% with nanoclay–dsRNA vs. 73.3% in the control at 120 hpi, effect visible up to 15 days; sporulation was strongly reduced	10-fold change	

Carbon quantum dots (CQDs)	synthetic, generally considered non-biodegradable	*P. infestans*	*in vitro* mycelial-growth assay	no dsRNA used	solid: 1.25, 2.5, 5, 10, 20 and 40 ng/µL liquid: 10, 20, 40, 80 and 160 ng/µL	/	at 9 dpi 40 ng/µL completely inhibited growth in solid medium; 40 ng/µL or higher reduced growth by 60-75% in liquid medium	/	(Kostov et al., 2022)
			*in vitro* developing-sporangium assay	*PiGpb1*	40 ng/µL dsRNA, 0.04 ng/µL CQDs	mass ratio 1:1000	inhibitory activity and 50% silencing for 72h	yes	
Star polymer (SPc) + cellobiose	SPc synthetic, cellobiose biodegradable	*P. infestans*	*in vitro* mycelium and spores	*eGfp*	naked dsRNA: 250 ng/µL	/	improved uptake of dsRNA using SPc:cellobiose after 12 h	no	(Wang et al., 2023c)
			potato and tobacco detached leaf assay	*eGfp* *PiHmp1* *PiCut3*	500 ng/µL dsRNA	mass ratio 1:1, 100 nm, cationic	Reduced lesion development and improved protection relative to naked dsRNA up to 12 dpi	1.5 fold change	
			potato plants in greenhouse and in the field		24 ng/µL dsRNA, 138 ng/µL cellobiose, 2760 ng/µL SPc	mass ratio 1:5.75:115, cationic	reduced infection and biomass in the greenhouse, 68 % protection at 29 dpi in the field (outperforming mancozeb)	no	
PEGDA functionalized carbon dots	synthetic	*P. capsici* *P. infestans* *P. sojae*	*in vitro* mycelium and spores	*CesA3* *Osbp1* *Gfp*	150 ng/µL dsRNA	mass ratio 1:12.5, cationic, 106.2 nm	carbon dots could facilitate dsRNA delivery in plant, reflecting in stronger fluorescence signal after 12h	no	(Wang et al., 2023d)
			tobacco or chili detached leaf and whole plant assay		200 ng/µL dsRNA		Strong disease suppression and improved performance relative to naked dsRNA for 7 days	yes (only for naked dsRNA)	
Membrane chimeric nanovesicles (EV–mimetic)	partially biodegradable	*P. infestans*	*in vitro* spores	*eGfp* *PiHmp1*	150 ng/µL dsRNA	mass ratio 30:600, cationic, 403.3 nm	enhanced intracellular delivery compared to naked dsRNA after 12h	no	(Zhang et al., 2024)
			tomato or cucumber detached leaf and whole plant assay		30 ng/µL dsRNA		enhanced uptake and fluorescent signal compared to naked dsRNA after 3 days, clear anti-infection effects at 5 dpi and decreased lesion area at 3 dpi compared to naked dsRNA	1 fold change	
Virus-like particles	biodegradable	*P. infestans*	foliar application	not mentioned	/	/	Improved disease suppression compared with naked dsRNA	not mentioned	(Boddu, 2025)
Cell-penetrating peptide (CPP) TAT	synthetic	*P. infestans*	*in vitro* spores	*Gfp*	200 ng/µL dsRNA	ratio N/P 2 cationic, 1.4 µM	Increased TAT uptake after 6h	no	(Kostov et al., 2026)
			tomato and potato detached leaf assay	*PiGpb1*	silencing: 450 ng dsRNA, 340 ng TAT uptake: 5-10 µM TAT		Reduced pathogenicity and less transcripts were found compared to naked dsRNA after 72 h, increased leaf uptake after 2h	40% reduction	
Gelatin and whey protein concentrate (WPC)	biodegradable	*P. capsici*	pepper seedling assay	*CesA3*	20 ng/µL OligoDNA	1.25 NM	disease index approximately 16% at the endpoint	no	(Delavari et al., 2026)

The table compiles target organism and target genes, carrier type and material class, nucleic acid cargo (e.g., dsRNA and DNA-based constructs), and reported effects on gene silencing and disease suppression, highlighting the rapid development of delivery strategies to overcome dsRNA instability and uptake barriers in oomycete pathosystems.

Among synthetic systems, star polymers (SPc) represent one of the earliest nanocarrier platforms explored for oomycete control. Initial studies employed SPc to deliver the plant defense elicitor chitosan rather than dsRNA, demonstrating enhanced induction of potato immune responses and effective suppression of late blight under both greenhouse and field conditions. These SPc-dsRNA formulations (17.4 nm), resulted in 30–40% disease suppression at 6 days post-infection (dpi) and up to 69.3% protection in the field (Wang et al., 2021). Building on this concept, subsequent work incorporated SPc into a self-assembled multicomponent nano-bioprotectant containing dsRNAs targeting the *P. infestans haustorial membrane protein 1 (PiHmp1)* and *cutinase 3 (PiCut3)*, together with the plant elicitor cellobiose. Although these SPc-dsRNA complexes were much larger in size (≈100 nm), they achieved improved lesion suppression up to 12 dpi and 68% protection at 29 dpi in the field—surpassing the well-used mancozeb (53%) (Wang et al., 2023c). This formulation improved dsRNA adhesion, uptake, and stability on potato leaves and provided superior disease control compared with SPc-delivered dsRNA alone or the conventional fungicide mancozeb in field trials, through the combined effects of RNAi-mediated pathogen inhibition and enhanced activation of plant defense responses (Wang et al., 2023c). Notably, SPc-based systems provided a strong protection window of up to 12 days, whereas naked dsRNA showed no protection after 24 hours, highlighting the importance of foliar retention and controlled release. These findings demonstrate how synthetic nanocarriers can be engineered to improve nucleic acid delivery while simultaneously integrating complementary disease control strategies to enhance efficacy under field conditions.

Nanoclay, an inorganic non-biodegradable carrier, has emerged as one of the most successful platforms for dsRNA delivery in *P. infestans*. Nanoclay–dsRNA complexes targeting genes such as *P. infestans sorbitol dehydrogenase* (*PiSdh*), *heat shock protein-90* (*PiHsp90*), *elongation factor 1-alpha* (*PiEf1α*), *phospholipase-D like 3* (*PiPld-3*), and *glycosylphosphatidylinositol-anchored acidic serine–threonine rich HAM34-like protein* (*PiGpi-Ham34*) consistently outperformed naked dsRNA, reducing pathogen growth, sporulation, and disease symptoms both *in vitro* and *in vivo* (Sundaresha et al., 2022; Sundararajan, 2023). Across systems, nanoclay was applied at concentrations of 2–50 ppm in combination with 200–250 ng/µL dsRNA, resulting in 70–90% reduction in mycelial growth at 10 dpi, reduced lesion development in detached leaves, and disease severity as low as 4% at 120 hpi. Notably, the protective effect persisted for up to 15 days, while transcript levels were reduced up to 10-fold, demonstrating both efficient dsRNA uptake and sustained RNAi activity. This enhanced efficacy is attributed to the ability of nanoclay to protect dsRNA from degradation, improve leaf adhesion, and facilitate the gradual release of the dsRNA cargo, demonstrating the value of controlled-release carrier architectures for achieving sustained RNAi activity (Sundaresha et al., 2022).

Carbon-based nanomaterials have also shown considerable promise. Carbon quantum dots (CQDs) enhanced dsRNA uptake and gene silencing efficiency targeting *P. infestans G-protein β subunit 1* (*PiGpb1*) and additionally exerted direct inhibitory effects on pathogen growth (Kostov et al., 2022). CQDs were applied at extremely low doses (0.04–40 ng/µL), with a mass ratio of 1:1000 (dsRNA: CQD), completely inhibiting mycelial growth at 40 ng/µL and achieving 50% gene silencing for 72 hours. Advances in carrier chemistry have led to functionalized carbon dots being applied across multiple *Phytophthora* species, including *P. infestans*, *P. capsici*, and *P. sojae*, to deliver dsRNAs targeting *cellulose synthase (Cesa3)* and oxysterol-binding protein *(Osbp)*. These carriers (≈106 nm) provided strong disease suppression for up to 7 days in tobacco and chili leaves through improved dsRNA stability, enhanced cellular entry, and efficient intracellular trafficking following 12 hours of exposure (Wang et al., 2023d). Their tunable surface chemistry makes them attractive candidates for pathogen-specific targeting strategies.

Biologically inspired carriers offer an alternative approach with improved environmental compatibility. Membrane-chimeric nanovesicles, designed to mimic EVs, efficiently encapsulated dsRNA and promote its protection, uptake, and systemic movement within plant tissues. Delivery of dsRNAs targeting *enhanced green fluorescent protein (eGfp)* or *P. infestans haustorial membrane protein 1 (PiHmp1)* significantly increased SIGS efficacy compared with naked dsRNA treatments in tomato and cucumber leaves. These vesicles were relatively large (403 nm), enhancing intracellular delivery after 12 hours and reducing lesion area at 3–5 dpi (Zhang et al., 2024). Similarly, virus-like particles (VLPs) offer a biodegradable encapsulation strategy that increases foliar delivery efficiency and disease suppression, although the target genes used in the study were not specified (Boddu, 2025).

Peptide-based carriers represent yet another promising design strategy. The cell-penetrating peptide (CPP) TAT, derived from the HIV-1 transactivator of transcription protein, markedly increased dsRNA uptake in *P. infestans*, resulting in stronger silencing of *Gfp* and *PiGpb1* and reduced pathogenicity in tomato and potato detached-leaf assays. TAT was applied at N/P 2 (1.4 µM), achieving 40% transcript reduction after 72 hours and increased leaf uptake within two hours, demonstrating rapid intracellular delivery compared with other carriers (Kostov et al., 2026). Such CPPs are particularly valuable for overcoming the cell wall and membrane barriers that limit dsRNA entry into oomycete cells (Kostov et al., 2026).

Finally, protein-based biodegradable carriers, such as gelatin and whey protein concentrate (WPC), have been successfully used to deliver DNA interference (DNAi) targeting cellulose synthase A (CesA3) in *P. capsici*, reducing both root and stem rot severity. These carriers were applied at 20 ng/µL oligoDNA and achieved a disease index of ~16%. Although these systems have not yet been extensively explored in *P. infestans*, they illustrate the potential of food-grade materials for cost-effective RNAi-based crop protection formulations (Delavari et al., 2026).

Despite these advances, nanocarrier-mediated dsRNA delivery still faces several carrier-specific challenges that constrain performance in the potato–*P. infestans* pathosystem. Most formulations exhibit poor penetration though plant surface barriers, as carrier–cargo complexes must traverse the cuticle, cell wall, and apoplastic space before reaching sites of infection (Wu and Li, 2022; Komarova et al., 2023). Even when successful apoplastic accumulation is achieved, symplastic or intracellular delivery often remains uncertain. Comparing across studies, carriers with strong cationic charge (e.g., SPc, CQDs, EV-mimetic vesicles) generally show better uptake in *Phytophthora*, whereas larger carriers (>300 nm) often exhibit slower penetration. Furthermore, carrier performance is strongly influenced by physicochemical properties such as size, ζ-potential, and hydrophobicity, limiting their transferability across crops and environmental conditions (Schwab et al., 2016).

Controlled release represents another bottleneck. Although many carriers effectively protect dsRNA from degradation, release can occur prematurely, too slowly, or incompletely, reducing the window for productive RNAi (Wu et al., 2025; Sede et al., 2026). For example, nanoclay provides protection up to 15 days, whereas SPc-based systems persist for ~12 days, and CQDs provide only short-term (≤72 hours) silencing. Additional constraints include formulation instability, potential phytotoxicity or ecological risks, and the persistent challenge of translating successes from controlled environments to field conditions. Environmental factors such as UV exposure, rainfall, fluctuating humidity, and heterogeneous canopy structure can substantially reduce dsRNA persistence and uptake (Mujtaba et al., 2021; Wu et al., 2025; Sede et al., 2026).

Several engineering strategies can mitigate these issues. Smaller or tunable carriers with enhanced foliar adhesion can improve transport across plant barriers, while responsive nanocarriers designed for cellular uptake and endosomal escape may strengthen symplastic delivery efficiency (Komarova et al., 2023; Wu et al., 2025). Likewise, systematic optimization of carrier size, surface charge, and application dose may reduce performance variability, whereas pH-responsive or biodegradable architectures could provide more predictable release kinetics (Sede et al., 2026). Field persistence may be further improved by incorporating protective materials that increase leaf retention and environmental shielding. At the same time, prioritizing biodegradable and low-toxicity formulations could alleviate safety concerns (Wu et al., 2025; Sede et al., 2026). A final consideration is the cost of production of the carrier and possibility for upscaling.

These challenges are particularly relevant for *P. infestans*, as nanocarriers are intended to compensate for the pathogen’s inherently poor uptake of exogenous dsRNA. Consequently, successful delivery depends not only on protecting dsRNA but also on ensuring that the carrier–cargo complex reaches the correct plant tissues and releases its payload before infection progresses. In this regard, the self-assembled multicomponent nano-bioprotectant developed by Wang et al. (2023c) highlights an important principle: the most effective systems are those combining foliar retention, plant penetration, and controlled release, rather than simply maximizing dsRNA binding capacity.

Taken together, these examples underscore both the diversity and potential of nanocarrier platforms for dsRNA delivery in *Phytophthora* species. They also reveal several key design principles that should guide future carrier development. Effective systems must protect dsRNA from UV radiation and nucleases, adhere strongly to the hydrophobic leaf surface, penetrate both the plant cuticle and oomycete cell wall, promote efficient endocytosis and endosomal escape, and release dsRNA in a controlled manner to sustain RNAi activity. Future improvements may be achieved through the incorporation of pathogen-responsive release mechanisms, surface ligands that recognize oomycete cell wall components, or carrier architectures that enhance apoplastic mobility. As material engineering advances, nanocarriers specifically tailored to the biological and ecological features of *P. infestans* are likely to become central to the development of robust, field-ready SIGS technologies.

## 4 From plant surface to oomycete: mechanistic insights

### 4.1 Uptake in the plant

Plant cells possess a highly dynamic endocytic machinery capable of internalizing a broad range of environmental molecules. Both clathrin-dependent and clathrin-independent pathways operate simultaneously, enabling uptake of membrane proteins, lipids, carbohydrates, and soluble extracellular components. These pathways are essential for nutrient acquisition, plasma membrane turnover, receptor internalization, and responses to environmental stimuli (Fan et al., 2015; Dragwidge and Van Damme, 2020).

The capacity of plant cells to internalize nanoscale particles was studied in detail by Bandmann et al. (2012), who showed that tobacco bright yellow-2 (BY-2) cells internalized nano beads through both clathrin-dependent (CDE) and clathrin-independent endocytosis (CIE). Uptake was strongly size-dependent and constrained by the presence of the cell wall. Nanobeads of 20 nm were readily internalized, whereas 100 nm beads were excluded from both turgid and plasmolyzed cells. Interestingly, removal of the cell wall by protoplastation enabled the uptake of substantially larger beads, in some cases reaching diameters of up to 1000 nm (Bandmann et al., 2012). These observations establish the plant cell wall as a major physical barrier that must be overcome by nanoparticle-based delivery strategies.

Once internalized, extracellular materials enter a complex endosomal network in which early endosomes and trans-Golgi network compartments regulate cargo trafficking, while multivesicular bodies coordinate sorting, recycling, and degradation (Muller et al., 2007; Robinson et al., 2008). Advances in live-cell imaging have greatly expanded our understanding of plant endocytic routes. Using pH-sensitive membrane dyes (e.g. FM4-64), endocytosis inhibitors (e.g. brefeldin A), and high-resolution microscopy, Dragwidge and Van Damme (2020) have shown that plant endocytosis is rapid, actin-dependent, and tightly integrated with cell wall remodeling and cytokinesis.

This intracellular membrane-trafficking network is highly relevant to the delivery of environmental RNA molecules. Any externally applied dsRNA, whether free or carrier associated, must navigate these endosomal compartments before reaching the cytoplasm, where the RNAi machinery resides and dsRNA processing occurs. Fluorescently labeled dsRNA has therefore become an important tool for visualizing uptake and intracellular movement in plants (Numata et al., 2014; Delgado-Martin et al., 2022; Qiao et al., 2023; Pal et al., 2024). Although uptake efficiency varies among plant species, tissues, and developmental stages, such variation is consistent with the differential endocytic capacities observed for other extracellular cargo molecules (Wytinck et al., 2020). Nanocarrier systems can improve the dsRNA uptake by promoting endocytosis and facilitating transport through the apoplast (Christiaens et al., 2020a).Their performance is largely influenced by physicochemical properties such as surface charge, particle size, and surface chemistry which affect interactions with both the plant cell wall and the plasma membrane (Christiaens et al., 2020a). Supporting this role, several endocytosis-related and vesicle trafficking genes, including *EH-domain-binding protein (StEpsin)*, *protein-sorting-associated protein 36 (StVPS36)*, *vesicle-associated membrane protein* (*StVamp1-2, StVamp3-1*) and *ras-related GTPase (StRab)*, were found to be upregulated in potato plants treated with cellobiose/SPc complexes during late blight studies (Wang et al., 2021, Wang et al., 2023c). These observations suggest that nanocarrier-mediated delivery may actively engage and stimulate endogenous membrane-trafficking pathways.

Following internalization, carrier-dsRNA complexes must release their cargo into the cytoplasm to enable interaction with the RNAi machinery. Several mechanisms have been proposed to explain this process, including intracellular pH changes or the proton-sponge hypothesis. The latter proposes that polymers rich in protonatable amine groups, such as dendrimers, buffer endosomal acidification and induce osmotic swelling, ultimately leading to membrane rupture and cargo release (Freeman et al., 2013). However, evidence from studies using polyethyleneimine (PEI) in human HeLa cells indicates that PEI does not necessarily raise endosomal pH, suggesting that endosomal escape may instead involve direct membrane interactions rather than classical proton buffering (Benjaminsen et al., 2013). Together, these studies indicate that endosomal escape mechanisms are dependent on carrier composition and biological context, and that proton-sponge activity should not be regarded as a universal mechanism.

Overall, current evidence suggests that successful dsRNA delivery in plants is governed not only by uptake at the cell surface but also by subsequent intracellular trafficking and endosomal escape. A deeper understanding of these processes will be essential for the rational design of nanocarriers capable of efficiently transporting dsRNA to the cytoplasm and maximizing RNAi efficacy in crop protection applications.

### 4.2 Uptake in *P. infestans*

In contrast to the relatively well-characterized mechanism of endocytosis in plants, the uptake of environmental or exogenously applied dsRNA in *P. infestans* remains poorly understood. Multiple studies have shown that *P. infestans* is capable of dsRNA uptake, but that this process is inconsistent and dependent on cell type, with substantially lower uptake rates than observed in many fungi and insects (Wytinck et al., 2020). Similarly, Sundaresha et al. (2022) and Sundararajan (2023) demonstrated that *P. infestans* sporangia can internalize fluorescently labelled dsRNA both *in vitro* and from sprayed leaf surfaces, supporting the capacity for environmental dsRNA uptake but without identifying a specific uptake mechanism. Furthermore, Qiao et al. (2021) observed that the efficacy of SIGS-mediated disease suppression across plant pathogens is strongly correlated with dsRNA uptake efficiency, with *P. infestans* consistently ranking among the least responsive species. This was recently confirmed in a comparative study assessing uptake of labelled dsRNA in diverse plant pathogens (Kostov and Smagghe, 2026). Collectively, these findings indicate that although dsRNA uptake occurs in *P. infestans*, it is neither robust nor uniform across developmental stages, including sporangia, zoospores, germ tubes, and hyphae.

Studies on dsRNA internalization in fungi and insects suggest that endocytosis is a major route for the uptake of RNA from the environment (Wytinck et al., 2020). While direct mechanistic evidence in *P. infestans* is currently lacking, the pathogen’s demonstrated ability to internalize environmental dsRNA implies that similar uptake pathways may operate, albeit with lower efficiency. Potential entry sites include the sporangial surface, where exposure to environmental dsRNA is greatest at germination sites, where cell wall remodeling may transiently increase permeability, and at haustorial interfaces, where intimate contact with host cells could facilitate cross-kingdom RNA transfer. However, none of these putative routes have been conclusively demonstrated. Consequently, inefficient uptake remains the principal biological bottleneck limiting SIGS efficacy in oomycetes. As a result, application of naked dsRNA frequently results in variable and sometimes weak disease suppression.

To address these limitations, several studies have explored nanocarrier-based delivery systems, as discussed in section *3.2, Overview of nanocarrier platforms applied to SIGS against P. infestans*. At present, there is no direct evidence that *P. infestans* employs a proton sponge-mediated mechanism for intracellular dsRNA release. Instead, the prevailing interpretation is that nanocarriers enhance SIGS performance primarily by improving dsRNA stability, leaf adhesion, and the probability of uptake by target cells.

In summary, RNA-based disease control through SIGS is biologically feasible in *P. infestans*, and naked dsRNA applications can induce measurable levels of gene silencing and disease suppression. However, the inherently low and variable uptake capacity of the pathogen limits the consistency and overall efficacy of this approach. Consequently, the most rational approach is to combine dsRNA with delivery systems that enhance stability, adhesion, and cellular internalization, thereby increasing the likelihood that the dsRNA reaches its intracellular target. Importantly, while dsRNA uptake in *P. infestans* remains inefficient and highly variable, plant cells are well documented to internalize extracellular dsRNA and nanocomplexes through multiple endocytic routes, including both CDE and CIE. The plant host therefore represents a more reliable and biologically accessible entry point for dsRNA delivery than direct uptake by the pathogen itself.

### 4.3 Communication between plant and *P. infestans*

Pathogen-targeting dsRNAs that are internalized by the plant cells can be processed by the plant RNAi machinery without affecting transcripts inside the plant. During infection, *P. infestans* rapidly colonizes the host tissues: following germination, zoospores or sporangia form appressoria, penetrate the epidermis, and subsequently establish haustoria. These specialized feeding structures invaginate the host plasma membrane without breaching it, creating a specialized biotrophic interface for nutrient acquisition and signal exchange (Wang et al., 2018). This interface provides a focal point for molecular exchange and may facilitate the transfer of RNA molecules—including free dsRNA, siRNAs, or EV-associated RNAs— from plant cells into the pathogen, providing a mechanistic basis for plant-mediated delivery of exogenous dsRNA. However, direct experimental evidence for RNA transfer into *P. infestans* remains limited. Understanding the mechanisms that govern plant–pathogen communication is essential for RNA-based disease control, as these natural exchange routes ultimately determine when and how dsRNA becomes accessible to the pathogen.

Haustoria also function as secretion hubs, enabling the delivery of apoplastic and intracellular effectors that manipulate host physiology and suppress immune responses. Transcriptomic analyses have shown that *P. infestans* dynamically reprograms its secretome during the transition between biotrophic and necrotrophic growth phases. This includes the production of adhesion factors such as cellulose-binding elicitor lectins, apoplastic protease inhibitors, and intracellular effectors that target host trafficking, immunity, and programmed cell death pathways (Gaulin et al., 2002; Tian et al., 2005; Zuluaga et al., 2016; Petre et al., 2021; Zheng et al., 2025). Together, these studies directly demonstrate that successful colonization depends on both apoplastic manipulation of the host environment and intracellular delivery of virulence factors.

Protein effectors represent the best-characterized communication channel between oomycetes and their hosts. A major breakthrough in understanding host–pathogen communication came from the discovery that *P. infestans* RXLR effectors enter plant cells through CDE. Wang et al. (2023b) demonstrated that silencing *clathrin heavy chain (NbCHC)* or the endosomal regulator *rab GTPase Ara6 (NbAra6)* in *Nicotiana benthamiana* markedly reduced internalization of *P. infestans* RXLR effectors and attenuated *P. infestans* colonization. The co-localization of RXLR effectors with endosomal markers provided direct evidence for CDE-mediated entry, resolving how extracellular RXLR proteins cross the plant plasma membrane. Building on this work, an immunoisolation-based approach was developed to allow purification of specific plant endosomal vesicles and their cargo during infection. This methodology allowed the co-isolation of RXLR effectors with endosomal compartments and provided a powerful tool for dissecting intracellular trafficking routes of pathogen-derived molecules (Wang et al., 2025a). Such advances are essential for distinguishing between host-mediated uptake, pathogen-mediated uptake, and extracellular persistence of biological macromolecules. Earlier conceptual studies had already proposed host-mediated endocytosis as a plausible route for effector entry in *P. infestans*, although direct experimental evidence was lacking at the time (Boevink et al., 2020). Similarly in other filamentous pathogens, endocytic pathways were found to be responsible for effector translocation (Wang et al., 2023a). Together, these studies establish host endocytosis as a central mechanism underlying protein effector entry into plant cells and suggest that similar cellular trafficking pathways may be contributing to the movement of RNA-based molecules.

In addition to proteins, RNA-mediated communication has emerged as a second major route of plant-oomycete interactions. Oomycete-derived sRNAs can associate with plant AGO/RISC complexes and modulate plant gene expression to promote virulence, demonstrating functional transfer of pathogen sRNAs into the host (Dunker et al., 2020). Conversely, *Phytophthora* species produce RNA silencing suppressors that counteract host RNA-based defense responses (Qiao et al., 2013). Evidence for bidirectional RNA exchange is further supported by the identification of pathogen-derived sRNAs predicted to target host immunity genes, including a *crinkler 1 (CRN1)*-derived sRNA proposed to target potato *enhanced disease susceptibility 1 (EDS1)* (Singh et al., 2026).

EVs provide an additional mechanism for communication across the plant-pathogen interface and may play an important role in the delivery of RNA molecules. EV-mediated exchange is well-documented in several plant–pathogen systems, but direct evidence in potato–*P. infestans* is still emerging. Recent studies highlight that EV-mediated communication involves both protein cargo and RNA cargo. For example, *P. capsici* releases EVs enriched in proteins associated with metabolism, transport, and pathogenicity that enhance zoospore virulence, while *P. infestans* EVs contain transmembrane proteins and RXLR effectors and can be distinguished from components of the EV-independent secretome through the use of *P. infestans membrane-derived protein 1 and 2 (PiMDP1-2)* as candidate EV markers (Fang et al., 2021; Breen et al., 2025). Moreover, *P. infestans* can actively manipulate plant EV trafficking during infection, further demonstrating possible EV involvement in host–pathogen interactions (Xu et al., 2026).

In addition to protein cargo, EVs also facilitate the movement of RNA molecules across the interface. Plant-derived EVs have been proposed to move through the haustorial interface and into *P. infestans*, providing a mechanistic basis for host-mediated transfer of exogenous dsRNAs and siRNAs (Palatinus, 2024). However, direct visualization or molecular confirmation of EV-associated RNA uptake by *P. infestans* has not yet been reported. Evidence for bidirectional RNA exchange is further supported by pathogen-derived sRNAs predicted to target host immunity genes, including a *P. infestans* CRN1-derived sRNA proposed to target potato *EDS1* (Dunker et al., 2020; Singh et al., 2026). These findings suggest that RNA molecules—both protein-bound and vesicle-associated—can move across the plant–oomycete interface.

Taken together, the literature reveals three converging communication modes—protein effectors, small RNAs and EVs —each of which exploits the same physical interfaces that define plant–oomycete interactions and all possible ways to affect transport of dsRNA from host plant to pathogen. The different possible routes through which dsRNA can be transferred from the host to *Phytophthora* are shown in **Figure 3**. Nanocarrier-assisted dsRNA delivery is designed to exploit and enhance these natural communication routes by enhancing RNA stability, uptake, and intracellular release. An overview of experimentally documented communication mechanisms across plant–oomycete systems is provided in **Table 3**.

**Figure 3:**
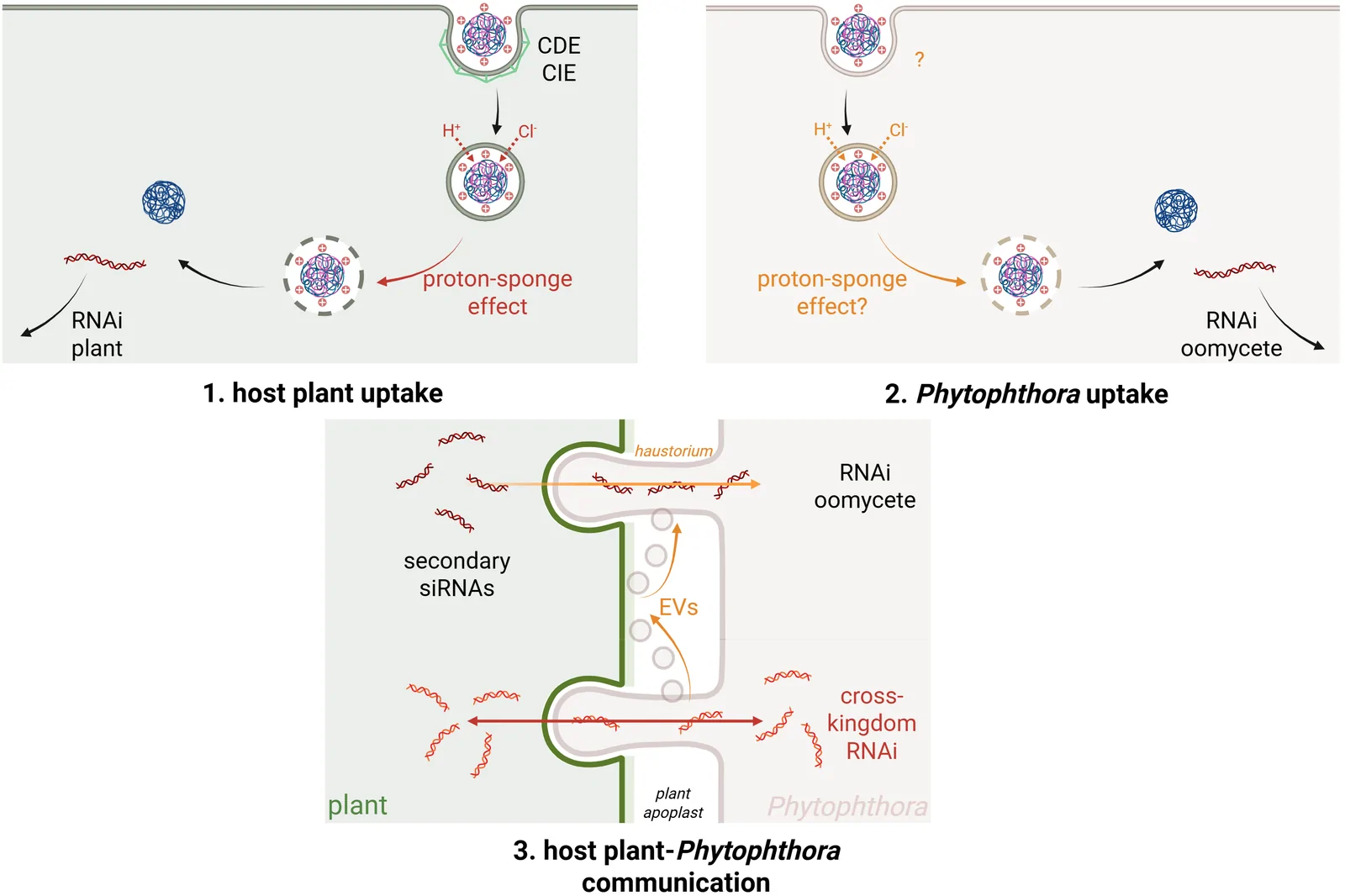
Schematic overview of the routes through which exogenously applied dsRNA or dsRNA–nanocarrier complexes can move from the plant to *Phytophthora* pathogens. The figure illustrates 1. plant-mediated uptake via cuticular penetration and endocytosis (CDE or CIE), followed by intracellular release and RNAi activation; 2. direct pathogen uptake of dsRNA or nanocarrier complexes by germinating spores or hyphae through endocytic or membrane-associated processes, followed by intracellular release and RNAi activation; and 3. infection-interface uptake, where *Phytophthora* encounters plant-associated dsRNAs and secondary siRNAs during colonization, as well as cross-kingdom RNA transfer from the pathogen to the host through haustoria, with EVs contributing additional RNA transport routes. Transport routes are differentiated according to their level of experimental support: black arrows indicate experimentally demonstrated pathways, red arrows indicate routes supported by indirect evidence, and orange arrows represent hypothesized mechanisms such as EV-mediated RNA transfer to *P. infestans*. Created in https://BioRender.com.

**Table 3:** Summary of plant–oomycete communication studies relevant to understanding natural and SIGS-mediated information flow.

Species/system	Communication mechanism studied	Key finding	Reference
*P. nicotianae*/plant interface	adhesion and cell wall interaction	CBEL is involved in cell wall deposition and adhesion to cellulosic substrates, supporting pathogen attachment, and host surface interaction	(Gaulin et al., 2002)
*P. infestans* – tomato	apoplastic effector–host protease interaction	a Kazal-like protease inhibitor from *P. infestans* inhibits and interacts with the tomato apoplastic PR protease P69B	(Tian et al., 2005)
Plant innate immunity	host communication/cell death regulation	autophagy regulates programmed cell death during the plant innate immune response	(Liu et al., 2005)
Oomycete pathogens	RNA silencing suppression	oomycete pathogens encode RNA silencing suppressors	(Qiao et al., 2013)
*P. infestans*	internal RNA biology linked to communication	tRNA fragmentation revealed active RNA silencing-related small RNA biology in *P. infestans*	(Asman et al., 2014)
*P. infestans* and potato	sRNA crosstalk and HIGS	sRNAs derived from effectors/TEs were identified; HIGS was successful, and potato resistance genes were targeted by native miRNAs	(Jahan, 2015)
*P. infestans* – tomato	infection-stage communication/transcript dynamics	transcript profiling showed strong stage-specific changes during hemibiotrophic infection, identifying infection-associated communication factors	(Zuluaga et al., 2016)
*Phytophthora* spp. – host plants	cross-kingdom RNAi/effector-mediated RNAi suppression	a *Phytophthora* effector suppresses trans-kingdom RNAi to increase disease susceptibility	(Hou et al., 2019)
Oomycetes – plant RNAi machinery	small RNA transfer/host RISC hijacking	oomycete small RNAs bind the plant RNA-induced silencing complex and contribute to virulence	(Dunker et al., 2020)
*P. capsici*	EV isolation and proteome	first characterization of EVs from *P. capsici*; EVs contained many proteins linked to metabolism, oxidation/reduction, transport, and pathogenesis, and adding EVs to zoospores increased virulence	(Fang et al., 2021)
*P. infestans* – host vesicle trafficking	effector targeting of host trafficking	host-interactor screens showed that vesicle trafficking is a major process targeted by *P. infestans* RXLR effectors	(Petre et al., 2021)
*P. infestans* – potato/*N. benthamiana*	cross-kingdom miRNA regulation	*P. infestans* Ago1-associated miRNA promotes potato susceptibility	(Hu et al., 2022)
*P. infestans* – *N. benthamiana*	effector uptake into host cells	RXLR effectors are taken up by host cells via CDE and accumulate in host endocytic vesicles	(Wang et al., 2023b)
*P. infestans* – potato	cross-kingdom miRNA/target regulation	potato and *P. infestans* miRNAs may regulate resistance and pathogen genes in both directions	(Luo et al., 2023)
*P. infestans* – Potato/*N. benthamiana*	cross-kingdom sRNA trafficking/EVs	sRNAs are transported in EVs; may mediate RNAi transfer in SIGS contexts	(Palatinus, 2024)
*P. infestans* – potato	intracellular effector-mediated immune suppression	Pi08174 suppresses potato immune defense through multiple resistance pathways	(Zheng et al., 2025)
*P. infestans*	EV proteome markers	EV-associated proteins included transmembrane proteins and RxLR effectors; EVs were distinct from the EV-independent secretome, and PiMDP1/PiMDP2 were proposed as EV markers	(Breen et al., 2025)
*P. infestans – N. benthamiana*	endosomal uptake/vesicle-mediated effector internalization	immunoisolation protocol to capture host endosomes and test whether secreted pathogen effectors enter plant cells via endocytic vesicles; useful for studying uptake and carrier-like transport routes	(Wang et al., 2025a)
Oomycetes – plant innate immunity	apoplastic host manipulation	oomycetes manipulate plant innate immunity through galacturonide oxidases	(Welsh et al., 2025)
*P. infestans* – potato	CRN-derived small RNA/host gene targeting	a CRN1-derived small RNA is predicted to target the potato immune regulator EDS1	(Singh et al., 2026)
Plant pathogens - foliar dsRNA	RNAi application/dsRNA transfer	foliar-applied dsRNA is mobile, transfers to plant pathogens, and triggers RNAi	(Brosnan et al., 2026)
*P. infestans* – plant interaction	EVs in infection	*P. infestans* targets plant EVs to promote infection, indicating EVs are part of the host–pathogen interaction interface	(Xu et al., 2026)

Entries specify the host–pathogen system, the communication mechanism investigated (protein effectors, apoplastic interactions, vesicle trafficking, cross-kingdom small RNAs, RNAi suppression, or dsRNA mobility), and their major findings.

## 5 European regulations and challenges for application

Within the European Union (EU), RNAi–based plant protection technologies are situated within a regulatory framework shaped by both established GMO legislation and the recent emergence of policies governing new genomic techniques (NGTs). HIGS falls under the strict framework of EU GMO legislation because it relies on transgenic plants engineered to express dsRNA. As a result, HIGS is subject to the authorization procedures and risk assessment requirements established under Directive 2001/18/EC (European Parliament and Council, 2001). In contrast, SIGS relies on the exogenous application of dsRNA molecules that do not involve genomic modification of the plant. As a result, SIGS is regulated within the existing framework for plant protection products, which focuses on evaluating environmental persistence, non-target effects, and exposure risks (Chen et al., 2025). This distinction is particularly important in the European context, where initiatives such as the European Green Deal and the Farm-to-Fork Strategy focus on the reduction in chemical pesticide use and promote the development of more sustainable and targeted crop-protection solutions (European Commission, 2020; Fetting, 2020). A SIGS-based approach would align seamlessly with the EU goals (Taning et al., 2020; De Schutter et al., 2022). At the same time, ongoing EU discussions on NGT regulation reflect a gradual shift toward a more nuanced biotechnology regulation, distinguishing transgenic approaches from targeted genome-editing technologies that do not introduce foreign DNA (EC Regulation 2026/1388) (European Commission, 2026). Although these regulatory developments do not directly apply to SIGS, they reflect a gradual modernization of biotechnology policy that may facilitate the future adoption of RNAi-based crop protection strategies.

The development of RNAi biopesticides is closely influenced by the selection of delivery materials, particularly the trade-off between synthetic and biodegradable carrier systems. Importantly, environmental safety must be evaluated at two distinct levels: the intrinsic safety of the dsRNA molecule itself and the environmental fate of the nanocarriers used to deliver it. A field study by Ghosh et al. (2026) examined the potato phyllosphere following SIGS targeting *P. infestans* and reported only limited, time-dependent shifts in bacterial communities and minimal disruption of dominant bacterial and fungal taxa. These results suggest that dsRNA applications exert relatively minor non-target effects compared to pathogen infection, but they also highlight the need to assess how different carrier chemistries may influence microbial dynamics, persistence, and ecological interactions over time. While dsRNA is rapidly degraded in soil and plant environments and exhibits high target specificity, nanocarriers may persist longer and interact with non-target organisms. Although naked dsRNA is generally biodegradable and quickly broken down by nucleases and environmental factors (such as UV radiation and microbial activity), nanocarrier formulations can substantially increase its persistence, leaf retention, cellular uptake, and target-site delivery (Wu et al., 2025). The extended stability of dsRNA complexes also increases the duration and spatial extent of exposure to non-target species, meaning that the environmental risk profile of a nanocarrier-based RNA pesticide cannot be inferred from dsRNA sequence specificity alone but must also consider the carrier’s physicochemical properties and environmental fate (Rodrigues and Petrick, 2020; Qiao et al., 2024). Synthetic nanocarriers, including PEI, dendrimers, and carbon dots, offer high loading capacities, tunable surface chemistry, and enhanced dsRNA stability. Their positive charge promotes strong complexation with dsRNA and can facilitate adhesion to plant surfaces and endocytic uptake. However, these same properties may cause carrier-dependent toxicity, as highly cationic materials can disrupt membranes, induce oxidative stress, and affect physiological processes in plants, insects, microorganisms, and other non-target organisms (Miyamoto and Numata, 2023). Nanocarriers may also indirectly influence off-target effects by prolonging dsRNA persistence or enabling its movement into tissues that naked dsRNA would normally not reach. Biodegradable carriers, including chitosan, lignin nanoparticles, and clay nanosheets, are generally more environmentally compatible as they degrade naturally after application. However, “biodegradable” does not necessarily mean risk-free: these materials can still affect microbial communities, modify dsRNA mobility, or release degradation products with distinct biological properties (Zarrabian and Sherif, 2025). Although these systems can provide effective delivery, their performance may be less consistent under field conditions, particularly in respect to dsRNA protection and controlled release (Qiao et al., 2024). Public perception tends to favor biodegradable and nature-derived materials. While RNAi technologies are often viewed as safer than transgenic approaches due to their specificity and lack of genome integration, societal acceptance remains a critical factor (Rinaldi et al., 2025; Tardin-Coelho et al., 2025). Transparent communication, evidence-based risk assessment, and clear demonstration of environmental benefits are essential for establishing public trust and ensuring a long-term social license to operate RNAi-based plant protection products.

Despite substantial advances in RNAi research, dsRNA-based biopesticides have not yet achieved widespread commercial adaptation. Production costs, delivery inefficiencies, and regulatory uncertainties continue to limit large-scale implementation. Current estimates indicate that dsRNA production and purification remain significant cost drivers, with experimental bacterial production systems reported at 43.2 €/mg, whereas scalable microbial platforms may reduce the costs to around 4$/g (3.65 €/g) (Nwokeoji et al., 2022). Given that field applications are estimated to require approximately 2–10 g dsRNA per hectare, delivery efficiency becomes a critical determinant of economic feasibility (Verdonckt and Vanden Broeck, 2022). Furthermore, environmental heterogenicity substantially affects performance. Temperature, humidity, leaf surface chemistry, UV exposure, and developmental stage of the pathogen can all influence dsRNA stability and RNAi success (Venu et al., 2024).

In addition to delivery-related constraints with respect to exogenous dsRNA/nanocarrier applications, target biology—including target function, infection-stage expression, and the extent of functional redundancy—can contribute to variability in RNAi outcomes. In *P. infestans*, the efficacy of exogenous dsRNA is strongly dependent on pathogen uptake, which varies among developmental stages and cell types. Therefore, both target selection and delivery must be optimized (Qiao et al., 2021). A promising target should contribute to pathogen survival, development, or virulence; be expressed when the pathogen is exposed to or receives dsRNA; occur in a nonredundant or sufficiently vulnerable process; be conserved among relevant *P. infestans* isolates; contain regions with limited sequence similarity to host and non-target organisms; and produce a phenotype when its transcript is only partially depleted. In a proof-of-concept SIGS study, dsRNAs targeting *PiGpb1*, *PiHmp1*, *PiCut3*, and *PiEndo3* reduced transcript abundance to different extents, whereas targeting *PiOsbp* resulted in lower transcript levels without a comparably strong disease phenotype, illustrating that molecular knockdown does not necessarily result in proportional biological control (Kalyandurg et al., 2021). The expression of target genes at a specific stage of infection further constrains the target choice because genes involved in zoospore germination, penetration, biotrophic colonization, the biotrophy-to-necrotrophy transition, and sporulation may be susceptible to silencing only during particular developmental windows (Zuluaga et al., 2016). In addition, dsRNA activity can depend on the selected transcript region and on sequence-specific properties such as target-site accessibility, predicted secondary structure, siRNA processing, sequence polymorphism, and isoform representation. Accordingly, testing multiple independent, non-overlapping dsRNA regions per gene is preferable rather than relying on a single fragment (Mosquera et al., 2025). Target identification is further complicated by incomplete functional annotation of the transcriptome and genetic variation among *P. infestans* populations (Haas et al., 2009; Lee et al., 2020). Candidate dsRNAs should therefore be screened both computationally and experimentally for potential homology to host transcripts and relevant non-target organisms. Collectively, these considerations indicate that effective RNAi-based control requires both efficient delivery and empirically validated target selection.

Regulatory requirements further slow commercialization because RNA-based plant protection products must undergo extensive environmental and ecological risk assessments before approval. Nevertheless, recent developments by Corteva Bioscience and GreenLight Biosciences demonstrate that industrial-scale dsRNA manufacturing and commercialization are technically feasible. GreenLight’s product portfolio includes Calantha™, the first commercial foliar-applied RNA insecticide, which recently obtained an emergency use authorization in Belgium, and Norroa™, which received U.S. EPA registration as a novel RNAi-based treatment against *Varroa* mites in honeybee colonies (Narva et al., 2025; Lester et al., 2026). These developments illustrate that RNAi biopesticides can successfully navigate regulatory pathways and enter commercial markets. However, the cost of these products, together with the continued need for extensive environmental data, highlight that affordability, delivery efficiency and long-term safety evaluation remain key bottlenecks for broader agricultural deployment.

Given these constraints, dsRNA-based technologies will likely be implemented alongside conventional fungicides rather than replacing them entirely, especially for high-pressure diseases such as late blight (Li et al., 2026). In such integrated strategies, conventional chemical agents provide rapid disease suppression, while RNAi introduces highly specific targeting of essential or virulence-related genes in the pathogen. This complementary mode of action creates opportunities to reduce overall chemical load while maintaining effective disease control (De Schutter et al., 2022).The different modes of action of the two strategies will also limit the emergence of pesticide resistance in the pathogen (De Schutter et al., 2022). Together, these economic, technical and regulatory factors help explain why dsRNA biopesticides have not yet achieved widespread agricultural adoption.

Advances in nanocarrier technologies are expected to address many of the limitations that currently remain. Indeed, the transformative potential of RNAi-based nanobiopesticides lies not solely in the use of a sequence-specific gene silencing mechanism, but in the rational design of delivery systems capable of maximizing stability, specificity, persistence, and biological efficacy in complex agricultural environments. Rather than functioning as passive carriers, next-generation nanocarriers are now being engineered as responsive delivery platforms that interact dynamically with both host plants and pathogens. One emerging concept is the development of pathogen-responsive nanocarriers that selectively release dsRNA in response to infection-associated stimuli such as pathogen-secreted enzymes, pH changes, or alterations in redox conditions (Da Silva et al., 2024; Mathur et al., 2025). Such systems enable localized activation of RNAi, concentrating the silencing effect at infection sites while minimizing off-target exposure and environmental loss. Parallel research efforts are focusing on multifunctional delivery systems that combine RNAi payloads with intrinsic antimicrobial properties. For example, by integrating dsRNA delivery with carrier materials that exhibit antifungal or anti-oomycete activity, these hybrid systems may provide synergistic protection through the simultaneous disruption of pathogen physiology and targeted gene silencing (Da Silva et al., 2024; Mathur et al., 2025). In addition, RNAi-based nanopesticides are increasingly being considered within the framework of precision agriculture. Their deployment could be integrated with disease forecasting models, sensor-driven monitoring networks, and drone- or robot-assisted spray technologies to enable spatiotemporally optimized applications. Such integration allows dsRNA formulations to be applied only when and where infection risk is highest, reducing input costs and environmental impact while maximizing protective efficacy.

Despite these promising developments, several critical challenges remain unresolved and warrant explicit consideration. Uptake mechanisms in both plants and oomycetes are still incompletely understood, with studies reporting conflicting evidence regarding the contribution of endocytosis, cuticular penetration, and extracellular stability. Independent studies report divergent uptake efficiencies across plant genotypes and environmental conditions, underscoring the need for standardized assays and cross-system validation (Fletcher et al., 2026). Similarly, SIGS efficacy varies substantially across experimental systems, pathogen isolates, and host genotypes, and only a limited number of independent field trials have validated laboratory findings. Contradictory results among studies, particularly regarding dsRNA persistence, uptake efficiency, and dose–response relationships—, highlight the need for standardized methodologies and cross-laboratory reproducibility. Part of this variability also reflects target-dependent factors, as RNAi efficacy can differ strongly between genes and even between transcript regions of the same gene (Mannaa et al., 2026). Nanocarrier safety also remains insufficiently characterized: while dsRNA itself is rapidly degraded, carrier materials differ widely in environmental fate, non-target interactions, and long-term accumulation. Economic constraints further complicated deployment, as formulation costs, production scalability, and durability under fluctuating field conditions continue to limit practical implementation. Together, these unresolved mechanistic, ecological, and economic uncertainties underscore the need for more rigorous, comparative, and field-based research before SIGS can be reliably integrated into mainstream crop protection.

## 6 Conclusion and future perspectives

Nanocarrier-enabled SIGS offers a compelling path toward next-generation control of *P. infestans*, combining the sequence-specific precision of RNAi with advanced delivery systems designed to overcome the biological and environmental barriers that have restricted RNAi efficacy until now. By protecting dsRNA from degradation, improving foliar adhesion, enhancing cellular uptake, and enabling targeted release at infection sites, nanocarriers transform SIGS from a fragile, short-lived intervention into a robust and field-compatible technology. The compatibility of these technologies with biodegradable materials and their alignment with non-GMO regulatory frameworks further strengthens their relevance within the European sustainability goals and integrated pest-management programs.

Future progress will depend largely on advances in delivery systems, including tunable or pathogen-responsive carriers, dsRNA formulations targeting multiple genes simultaneously, and multifunctional platforms that combine RNAi with intrinsic antimicrobial activity. As these technologies mature, nanocarrier-enabled SIGS is poised to shift from proof-of-concept studies to practical agricultural application, complementing resistant cultivars and reducing reliance on conventional fungicides. Integrating delivery innovation with rigorous environmental fate assessment will be essential for ensuring that SIGS technologies mature not only in efficacy but also in ecological and societal acceptability. Ultimately, this approach provides a realistic and environmentally aligned route toward more precise, adaptive, and durable late blight management—positioning RNAi-based technologies as a cornerstone of future crop protection rather than a niche alternative.
